# Combined Effect of Hadron Therapy and PCSK9 Inhibition Therapy on the Immune Response in STK11-Deficient Lung Adenocarcinoma

**DOI:** 10.3390/ijms27219778

**Published:** 2026-10-30

**Authors:** Elena Gantsova, Irina Arutyunyan, Anna Soboleva, Viktoriia Mikhailovskaia, Evgeniya Kananykhina, Daria Artemova, Kirill Mironov, Polina Vishnyakova, Andrey Elchaninov, Xuhui Bao, Andrey Makarov, Konstantin Gordon, Timur Fatkhudinov

**Affiliations:** 1 Research Institute of Molecular and Cellular Medicine, Peoples’ Friendship University of Russia (RUDN University), 6 Miklukho-Maklaya Street, Moscow 117198, Russia; labrosta@yandex.ru (I.A.); annasobo@mail.ru (A.S.); rybachuk251@gmail.com (V.M.); artiomova.darya@yandex.ru (D.A.); ksmironov@gmail.com (K.M.); vishnyakova-pa@rudn.ru (P.V.); elchandrey@yandex.ru (A.E.); makarov-av@rudn.ru (A.M.); gordonkb@nmicr.ru (K.G.); fatkhudinov-tkh@rudn.ru (T.F.); 2 Petrovsky Russian Scientific Center of Surgery (RSCS), 3 Tsurupa Street, Moscow 117418, Russia; e.kananykhina@gmail.com; 3 A. Tsyb Medical Radiological Research Center (MRRC), Federal State Budgetary Institution “National Medical Research Radiological Centre”, Ministry of Health of the Russian Federation, 4 Koroleva Str., Obninsk 249036, Kaluga Region, Russia; 4 Department of Oncology, Shanghai Pudong Hospital, Fudan University Pudong Medical Center, 2800 Gongwei Rd, Shanghai 201399, China

**Keywords:** lung adenocarcinoma, non-small cell lung cancer, serine-threonine kinase 11, hadron therapy, PCSK9

## Abstract

Lung cancer is the most common type of cancer worldwide. Tumors carrying a mutation in the STK11 gene are associated with severe prognosis and resistance to therapy. New treatments for pulmonary adenocarcinoma highlight the efficacy of High-LET neutron therapy, often combined with drug therapy. PCSK9, whose inhibitors are used to treat dyslipidemia, may also be a new target in lung cancer therapy. CRISPR-Cas9-mediated STK11 mutation was performed in murine LLC1 lung adenocarcinoma cells. Human A549 cells that naturally carry a homozygous loss-of-function mutation in the STK11 gene were also used. Cells were irradiated with protons, neutrons, and photons (2 Gy) and analyzed for survival, apoptosis, clonogenic survival assay, and evolocumab (PCSK9 inhibitor) sensitivity. In vivo xenograft (Nude) and syngeneic (C57BL/6) subcutaneous tumor models were neutron-irradiated (5 Gy). Tumor cell proliferation, microenvironment, and protein expression were assessed. High-LET irradiation confirmed the STK11-deficient lines radio resistance, which did not reduce under PCSK9 inhibitor treatment conditions in vitro. In animal models, the PCSK9 inhibitor decreased Ki67 index of tumor cells and infiltration of cancer-associated fibroblasts and macrophages in the combination therapy group. Particle therapy resistance serves as a crucial prognostic marker in the management of lung adenocarcinoma. Using cell lines and animal models, we successfully described this resistance phenotype and demonstrated the promising use of a PCSK9 inhibitor to enhance the efficacy of hadron radiotherapy against lung cancer.

## 1. Introduction

Lung cancer is the most prevalent malignancy worldwide and a leading cause of cancer-related deaths. As the leading cause of cancer mortality worldwide, lung cancer presents a significant global health burden. Its poor prognosis is reflected in a 5-year survival rate of just 10–20%. Most lung cancer diagnoses (80%) are of the non-small cell type (NSCLC), making it far more prevalent than small cell lung cancer (SCLC), which occurs in 20% of cases [1]. While searching for new therapeutic targets against lung cancer Dr. Bao’s research team identified [2] the involvement of PCSK9 in the progression of lung adenocarcinoma (LUAD) in 2020 [3]. PCSK9 (proprotein convertase subtilisin/kexin type 9) is a serine protease primarily expressed in the liver. It plays a key role in cholesterol homeostasis—specifically, in regulating levels of low-density lipoproteins (LDL) in the blood. Three clinical-grade PCSK9 inhibitors are now available: monoclonal antibodies (evolocumab, alirocumab) and siRNA (inclisiran), all used to lower LDL-C [4,5]. This investigation has provided an important basis for the development of PCSK9 inhibitory immunotherapy (PCSK9-it) as a potential comprehensive therapeutic strategy for STK11/LKB1-knockout LUAD, including particle therapy, a modern approach for tumor therapy [6]. Serine-threonine kinase 11 is expressed in all tissues and is involved in the regulation of cellular metabolism, energy homeostasis, cell polarity and migration, and acts as a tumor suppressor. Therefore, the development of effective therapeutic methods against STK11/LKB1-mut tumor resistance mechanisms is of great importance. Mutations in the serine-threonine kinase gene STK11 are associated with more aggressive tumor progression and resistance to particle therapy, leading to a higher rate of local recurrence and a worse clinical prognosis [7]. Clinical evidence indicates that STK11/LKB1 mutations contribute to resistance to both immune checkpoint inhibitors (ICIs) and radiotherapy (RT) in NSCLC, worsening prognosis and increasing local recurrence rates [8,9]. Mechanistically, STK11/LKB1 loss suppresses anti-tumor immunity by preventing immune effector cell infiltration into the tumor immune microenvironment (TIME), driving immune escape [10]. Hadron therapy (particle therapy), i.e., the use of protons or neutrons to treat tumors, can be used for this purpose [11,12]. Compared to conventional X-rays and gamma rays, particle therapy (PT) demonstrates high biological effectiveness while delivering low radiation doses to normal tissues. This enables particle radiotherapy to significantly improve the efficacy of lung cancer treatment while reducing side effects, particularly in locally advanced and recurrent NSCLC. Early-stage proton therapy for NSCLC has shown a 3-year recurrence-free survival rate of up to 80% in multiple studies. One retrospective study reported similar outcomes for both proton and carbon-ion therapy [13]. In locally advanced NSCLC—characterized by high mortality and therapy resistance—proton therapy resulted in lower rates of pulmonary, esophageal, and hematological toxicity compared to photon therapy [12]. High-linear energy transfer (high-LET) radiotherapy, delivered via neutrons and carbon ions, exerts multiple distinct biological effects on tumor tissues compared to conventional radiotherapy. By analyzing TCGA RNA-seq data for LUAD, we demonstrated that proprotein convertase subtilisin/kexin type 9 (PCSK9) expression is significantly elevated in tumor samples from patients with lung adenocarcinoma harboring STK11/LKB1 mutations. Furthermore, LUAD patients with low PCSK9 expression exhibit significantly better overall survival (OS) and progression-free survival (PFS) than those with high PCSK9 expression [14].

Despite advances in targeted therapies, the mechanisms underlying resistance to high-linear energy transfer (high-LET) radiotherapy in STK11-mutant lung adenocarcinoma remain poorly understood, and the potential of combining such radiotherapy with PCSK9 inhibition therapy (PCSK9-it) is entirely unexplored. It is crucial for advancing personalized oncology, as it could uncover novel strategies to overcome therapy resistance in a high-risk patient subgroup with dismal outcomes. The primary aim of this study is to investigate presence of radiotherapy resistance in STK11-mutant lung adenocarcinoma and evaluate the efficacy of combining high-LET neutron therapy with PCSK9-it. Specific objectives include establishing STK11-deficient cell lines; assessing their response to different radiation types (protons, neutrons, photons); evaluating the effect of a PCSK9 inhibitor on radiotherapy response in vitro and in vivo; and analyzing changes in tumor proliferation and the tumor microenvironment. This combinatorial approach as a novel therapeutic strategy for STK11-mutant LUAD patients.

## 2. Results

### 2.1. STK11 Mutation Confers Resistance to PCSK9-It and Particle Therapy in Lung Adenocarcinoma Cell Lines

**Figure 1:**
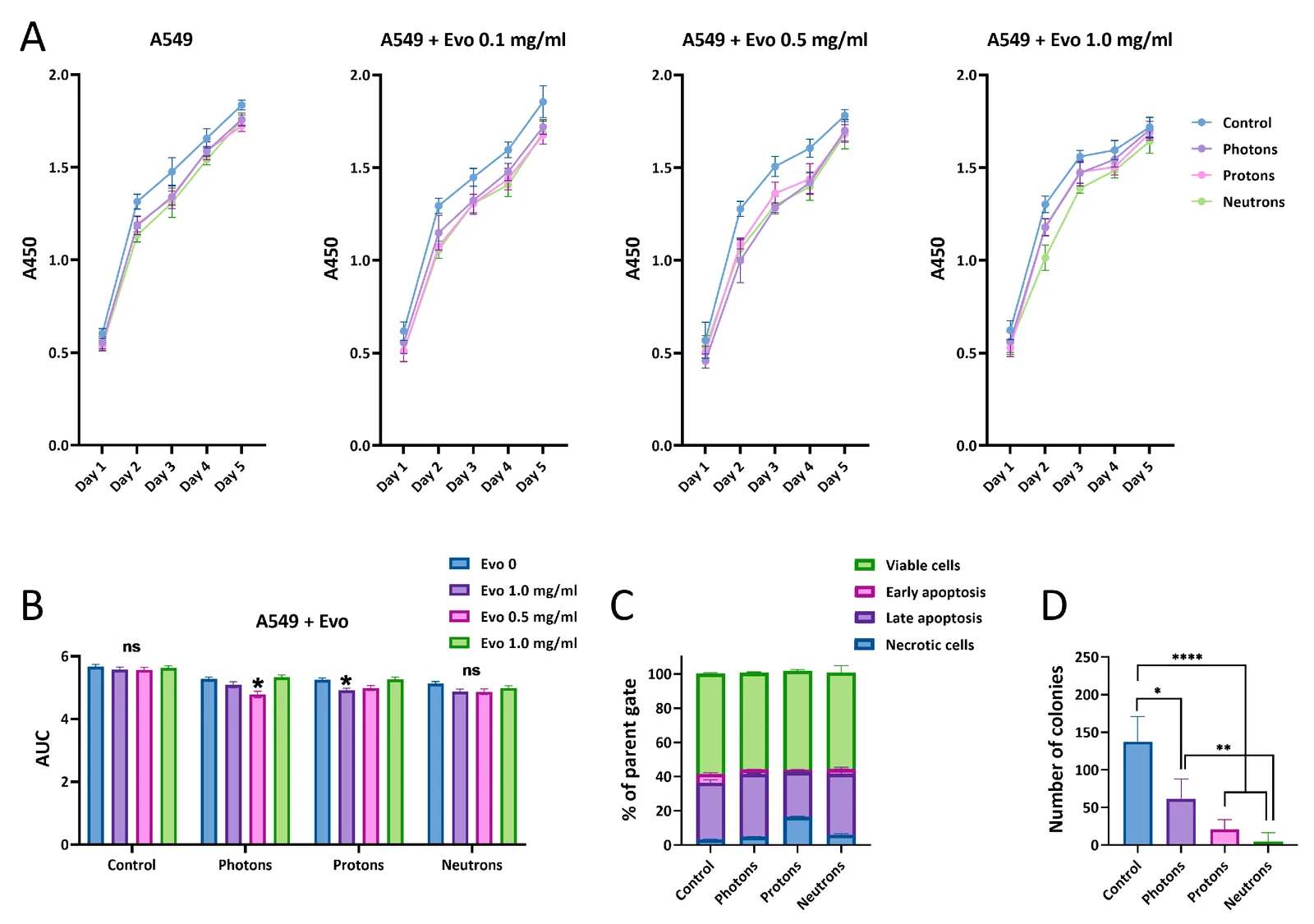
Effect of particle therapy and PCSK9-it on the human LUAD A549 cell line (*n* = 6 for each group). (**A**) CCK8 cell viability test at different inhibitor concentrations during 120 h after 2 Gy irradiation. (**B**) Area under the curve (AUC) analysis for CCK8 test (* *p* < 0.05, ** *p* < 0.01, **** *p* < 0.0001); (**C**) annexin V-PI test to determine the proportion of apoptotic cells; (**D**) colony-forming capacity after irradiation at a dose of 2 Gy. Data are presented with interquartile range and Kruskal–Wallis statistical test with Dunn’s post hoc test.

The production of a STK11-mutant Lewis lung carcinoma mouse line and its sensitivity to particle radiotherapy was described previously [15]. Human A549 cells that naturally carry a homozygous loss-of-function mutation in the STK11 gene were kindly provided by colleagues from A.B. Malashicheva’s laboratory at the Institute of Cytology of the Russian Academy of Sciences. Irradiation with photons (as a reference) or protons and neutrons (particle therapy) was carried out at the A.F. Tsyb Medical Radiological Research Center in Obninsk. A standard dose of 2 Gy was chosen as the radiation dose, which is usually used for patients as a single dose, and is also used in other studies on cell lines [16,17,18]. All exposure doses used in our experiment were biologically equivalent. A relative biological effectiveness (RBE) for protons was 1.1, and for fast neutrons RBE was 3. LUAD cell line viability 3 h after irradiation was high, being at least 95%. Thus, irradiation at a dose of 2 Gy did not lead to immediate death of A549 cells, which makes it possible to study the delayed biological effects of radiotherapy. Twenty-four hours before irradiation, PCSK9 inhibitor evolocumab was added to the cells at a concentration of 0.1, 0.5, or 1.0 mg/ml [19]; no inhibitor was added in the control group. Cell viability was assessed for 120 h, with the CKK8 test performed every 24 h (Figure 1A). The neutron therapy had the strongest effect, which is expected, as neutrons have higher Linear Energy Transfer (LET) and cause greater DNA damage, leading to cell death [13]. In Figure 1B, comparing the area under the curve, AUC, for concentrations of 0.1 and 0.5 mg/ml, a decrease in cell viability was observed after irradiation compared to the control, indicating a combined effect of the PCSK9 inhibitor and radiotherapy (Figure 1B). To identify the most effective type of hadron therapy, an assessment of apoptosis using annexin V-PI staining and colony formation assay was performed on Petri dishes. Figure 1C shows that protons significantly increase the proportion of necrotic cells. Figure 1C shows that cells irradiated with neutrons form fewer colonies, followed by approximately equal effects of protons and photons. Thus, neutron irradiation reduces the ability of LUAD cells to form colonies.

### 2.2. Evaluation of Combined PCSK9-It and Neutron Therapy in a Xenograft Model

**Figure 2:**
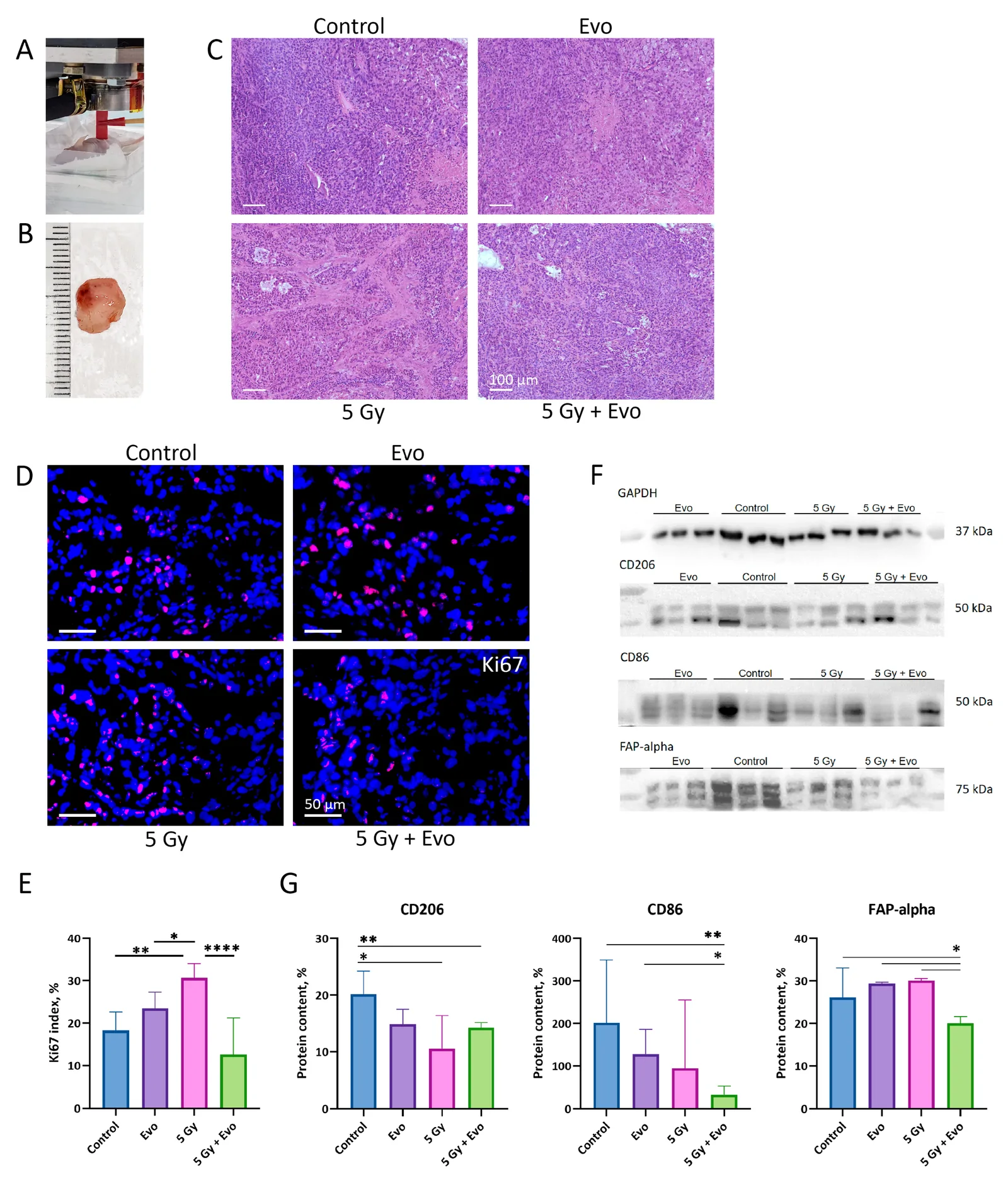
Cell-derived xenograft model on nude mouse with human LUAD. (**A**) Photo of particle therapy of nude mouse (*n* = 5 for each group) with 5Gy dose of neutron irradiation; (**B**) tumors of human LUAD; (**C**) histology of tumors stained with hematoxylin–eosin, 40×, scale marker 100 µm; (**D**) IHC Ki67 marker of proliferation in tumors of human LUAD; (**E**) Ki67+ index in control group, PCSK9-it group, 5Gy group and combination group (* *p* < 0.05, ** *p* < 0.01, **** *p* < 0.0001); (**F**) Western blotting of CD206, CD86 and FAP-alpha proteins in tumors lysates, where GAPDH were used as reference protein marker; (**G**) protein content of CD206, CD86 and FAP-alpha proteins (* *p* < 0.05, ** *p* < 0.01). PCSK9-it caused a significant decrease in both CD206 and CD86 macrophages. Data are presented with interquartile range and Kruskal–Wallis statistical test with Dunn’s post hoc test.

Tumors were modeled using subcutaneous injection of A549 cells at the Petrovsky Russian Scientific Center of Surgery. Animal weight and tumor growth were measured during the study. PCSK9-it was administered every 3 days at a concentration of 10 mg/kg. Seven days after irradiation (Figure 2A), the tumors were removed (Figure 2B) and stained with hematoxylin and eosin. The sections in Figure 2C show morphological changes reflecting key pathogenetic aspects of tumor growth. A well-developed collagen fiber network actively penetrates the tumor, creating a rigid stromal framework. The tumor parenchyma is characterized by pronounced cellular polymorphism and is represented by numerous atypical tumor cells with hyperchromic and polymorphic nuclei. Another feature is the disruption of the normal tissue histoarchitecture, manifested by the loss of cell polarity and a chaotic pattern of their arrangement. One of the potential prognostic markers in lung cancer is Ki67. Ki67 is widely used as a proliferation marker, which is predominantly detected during the active phases of the cell cycle, i.e., G1, S, and G2, as well as mitosis. In tumor tissue samples of the xenografted in vivo model, moderate proliferative activity of cells was confirmed (Figure 2D): the proportion of Ki67+ cells in control samples was 18.8 ± 5.6%. The addition of a PCSK9 inhibitor did not significantly affect the Ki67 index. However, a single exposure to neutrons at a dose of 5 Gy was associated with an increase in the Ki67 index to 31.5 ± 3.3% at a single endpoint. This may reflect compensatory proliferation, selection of proliferative cells, altered tumor composition, or stress-associated changes in cell state, rather than true stimulation of proliferation. At the same time, no increase in the Ki67 index was observed in the irradiated group “5 Gy + evolocumab” (Figure 2E). In a Western blot analysis (Figure 2F) of the FAP-alpha (Fibroblast Activation Protein Alpha protein), which is a marker of tumor-associated fibroblasts that make up up to 90% of the stroma of many solid tumors, xenograft models of tumor growth revealed that a significant decrease in the production of this protein was observed in combination with PCSK-it and 5 Gy radiation, compared to other groups, and that the content of macrophages in general was significantly reduced. Thus, the combination of PCSK9-it and neutron therapy enhance the antitumor effect.

### 2.3. Impact of Syngeneic Models on Macrophage Populations in Combined Therapy with PCSK9-It and Neutron Exposure

**Figure 3:**
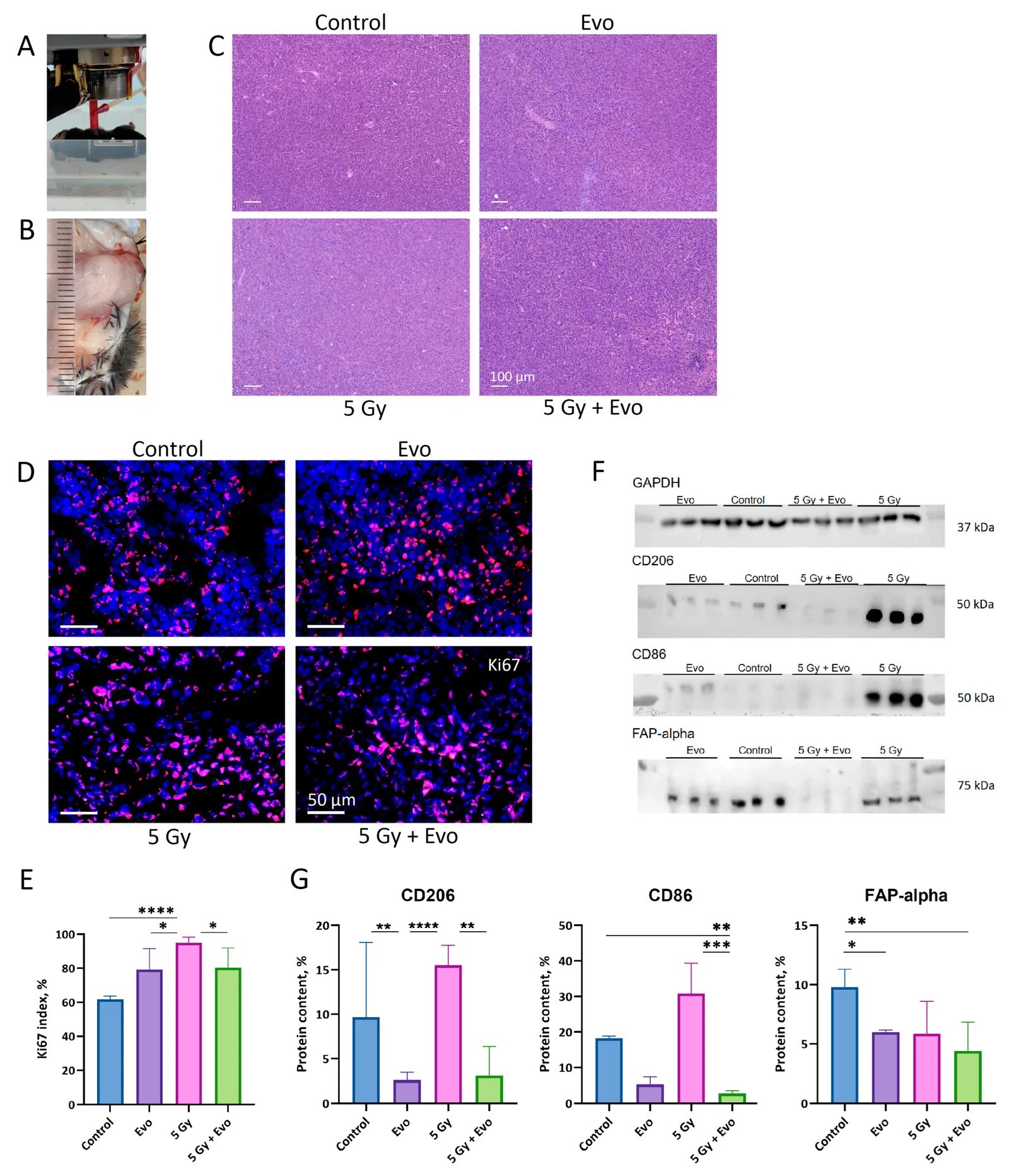
Allograft model on C57BL/6 mouse with LLC1 cells with STK11-Mut. (**A**) Photo of particle therapy of C57Bl/6 mouse (*n* = 5 for each group) with 5Gy dose of neutron irradiation; (**B**) tumors of LLC1-STK11-Mut; (**C**) histology of tumors stained with hematoxylin–eosin, 40×, scale marker 100 µm; (**D**) IHC Ki67 marker of proliferation in tumors LLC1-STK11-Mut; (**E**) Ki67+ index in control group, PCSK9-it group, 5Gy group and combination group (* *p* < 0.05, **** *p* < 0.0001); (**F**) Western blotting of CD206, CD86 and FAP-alpha proteins in tumor lysates, where GAPDH were used as reference protein marker; (**G**) protein content of CD206, CD86 and FAP-alpha proteins (* *p* < 0.05, ** *p* < 0.01, *** *p* < 0.001, **** *p* < 0.0001). PCSK9-it caused a significant decrease in both CD206 and CD86 macrophages. Data are presented with interquartile range and Kruskal–Wallis statistical test with Dunn’s post hoc test.

Tumors were modeled by subcutaneous injection of LLC1 STK11-mut cells, animal weight and tumor growth were measured, and PCSK9-it was administered once every 3 days at a concentration of 10 mg/kg. After a single dose of 5 Gy neutron irradiation (Figure 3A) tumors were isolated (Figure 3B). Analyzing the tumor sections stained with hematoxylin and eosin, we see tumor parenchyma with numerous foci of infiltration with a predominance of lymphocytes and macrophages (Figure 3C), reflecting an attempt at an antitumor immune response. In different study groups we see similar histoarchitecture, where the changes are probably at a deeper level, which we confirm with further research. Describing tumor proliferation using Ki67 IHC staining (Figure 3D) revealed high proliferative activity in the control group (61.5 ± 2.8%). Similar to the xenograft model described above, the addition of evolocumab did not significantly affect the Ki67 index, but a single dose of 5 Gy of neutron irradiation significantly increased the index to 94.7 ± 3.6%. At the same time, in the irradiated group “5 Gy + evolocumab”, the Ki67 index did not differ from the control (Figure 3E). We found that PCSK9-it suppresses the process of radio-induced proliferation of tumor cells, significantly reducing the Ki67 index in xenograft and allograft models of lung cancer. The effect of mitigating the radio-induced proliferative activity of cancer cells requires further study. When analyzing the relative content of proteins in the tumor after therapy in syngeneic models of tumor growth, in evolocumab FAP-α production was reduced compared to animals that did not receive the drug. A similar effect was observed when evolocumab was combined with particle therapy. PCSK9-it caused a significant decrease in both CD206 and CD86 macrophages (Figure 3F,G). The decrease in FAP-α levels indicates that the activity or number of tumor-associated fibroblasts was inhibited by the combined therapy.

### 2.4. Tumor Microenvironment (TME) and Immune Populations in Both Models Have an Impact of PCSK9-It and Hadron Therapy

**Figure 4:**
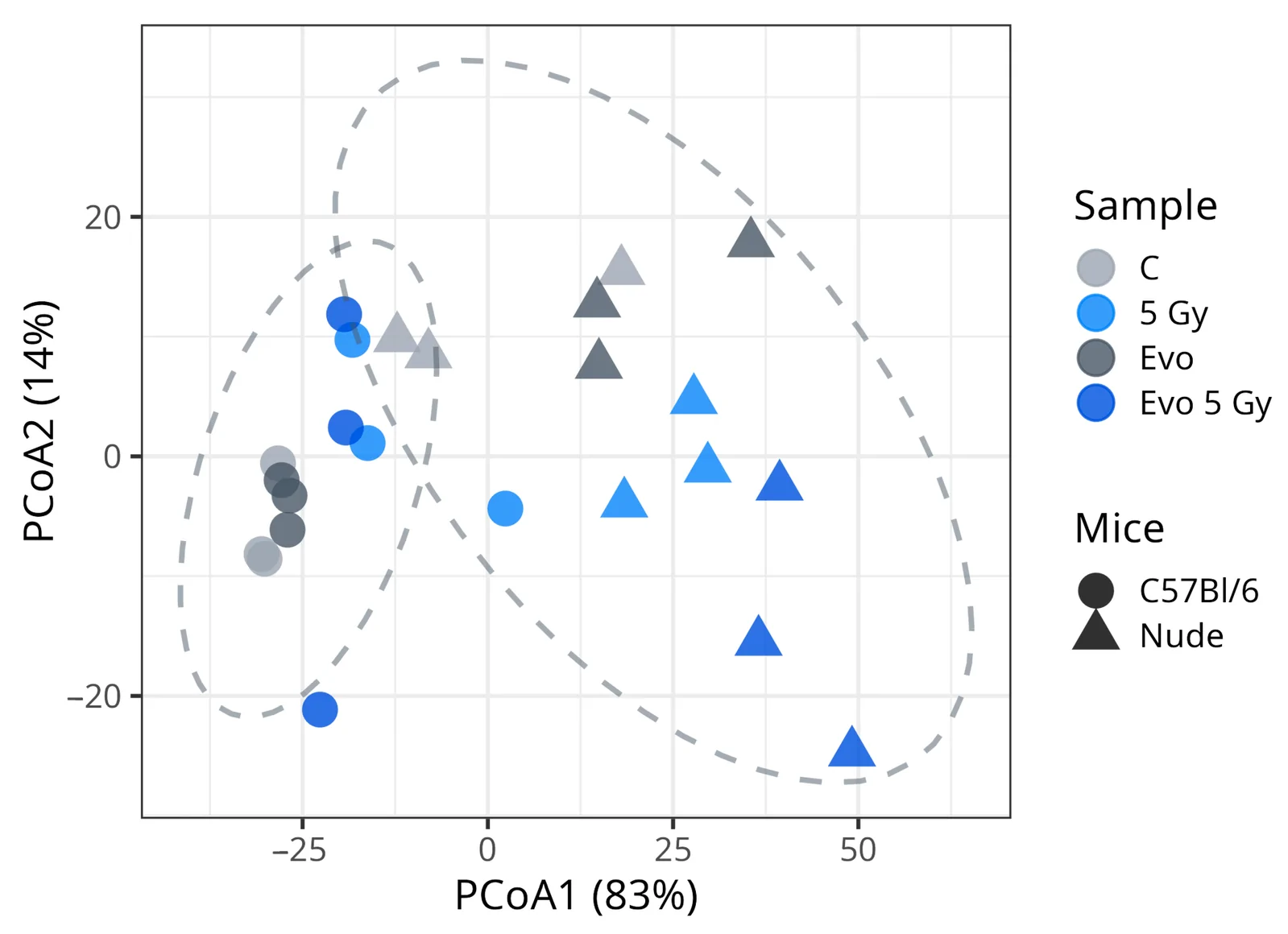
Three-factor topological profile of cell population distribution in C57Bl/6 and nude mouse tissues under combined therapy. Ordination was performed via Principal Coordinate Analysis (PCoA) based on a Euclidean distance matrix. The latent coordinate PCoA1 (83%) reflects a clear sample segregation based on the genotypically determined immunological status of the animals, while PCoA2 (14%) determines the vector of tissue-specific changes induced by the treatment (*Sample*). Dashed ellipses indicate the 95% confidence regions for the genetic strains (*Mice*). Global multivariate analysis of sequential effects was performed using PERMANOVA with 999 free permutations: the contribution of the *Mice* factor was 60% (*p* = 0.001), *Sample* accounted for 16% (*p* = 0.003), the non-linear *Mice* × *Sample* interaction contributed 11% (*p* = 0.009), and residual noise accounted for 13%. Sample size *n* = 3 per experimental group.

Using flow cytometry with antibodies specified in the Supplement Table S1, we analyzed the immune microenvironment cells of the tumor, peripheral blood mononuclear cells (PBMCs), and spleen immune cells, with gating strategies presented in Supplementary Figure S1. We analyzed immune-cell markers in both models to assess changes in immune-cell populations. To do this, we compared all groups using the three-factor tissue-specific profile analysis and PCoA Ordination. A global multivariate structure analysis using sequential PERMANOVA (by = “terms”) revealed a highly significant orthogonal contribution of the host genotype, establishing the *Mice* factor as the primary driver explaining 60% of the total variance (*p* = 0.001). The second factor, therapeutic treatment (*Sample*), explained 16% of the variance (*p* = 0.003). Crucially, the combined interaction effect of these factors (*Mice* × *Sample*) accounted for 11% of the total system variability and demonstrated high statistical significance (*p* = 0.009). The cumulative predictive power of the constructed multivariate model reached *R*^2^ = 0.87, leaving only 13% of the variance to random intra-group variability (residuals). This pronounced non-linear interaction effect provides strong evidence that the tissue-specific response vector of cellular populations to combined therapy is fundamentally modulated by the baseline immunological status of the organism. The horizontal axis effectively represents the dominant biological vector of changes associated with the immunodeficient status of the animals. The ordination plot displays a strict separation; C57Bl/6 are localized exclusively in the left half-plane, whereas nude mouse samples are clustered entirely in the right portion (Figure 4). This pattern aligns perfectly with the PERMANOVA results, where the genotype factor describes most of the variance (60%). The vertical axis describes the secondary vector of variability, reflecting a directional shift in intra-tissue *CD45+* cell pools under the influence of therapeutic agents (*Sample*) while preserving the general framework of genotype segregation. Such a high percentage of information retention indicates that the constructed two-dimensional projection reflects the true biological distances between objects with minimal distortion (see Figure 4). A comparative analysis of model predictive accuracy using the LOO-CV criterion confirmed the superiority of the robust Student’s *t*-distribution model over the classical Gaussian model, indicating the successful mitigation of isolated extreme values (outliers) in the raw data.

**Figure 5:**
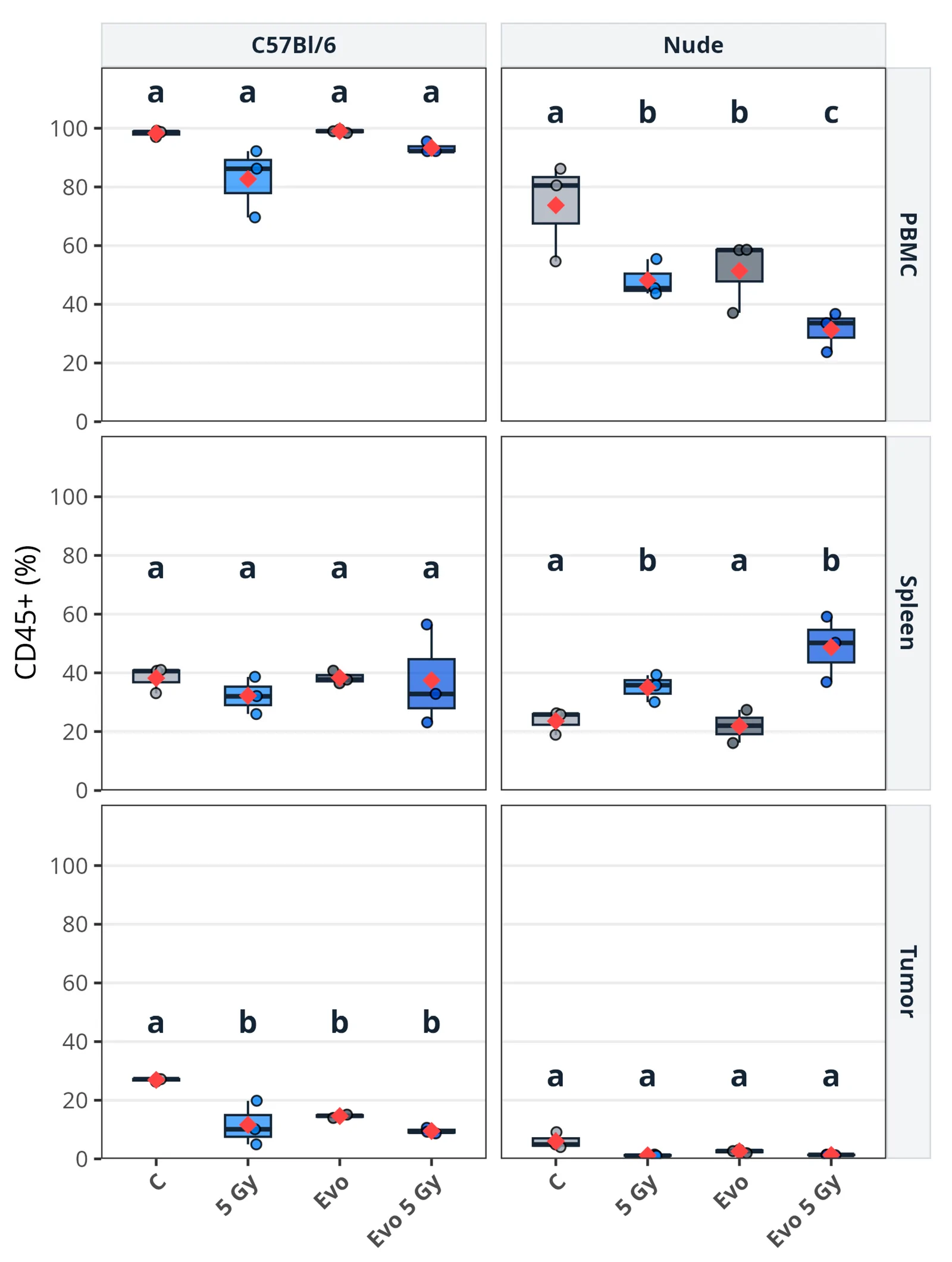
Statistical significance profile of cell distribution across experimental groups based on Bayesian CLD analysis. Boxplots display the medians, 25th, and 75th percentiles of the *CD45+* cell pool distribution (%), along with individual empirical values (blue and gray dots). Red diamonds indicate calculated posterior group means. Different lowercase letters (a, b, c) above the plots denote groups with credibly distinct posterior distributions (based on contrasts of group means, *p* (difference ≠ 0) > 0.95). Panels are faceted by mouse strain (C57Bl/6 and Nude, vertically) and tissue/organ type (PBMC, Spleen, Tumor, horizontally). Control “C”—intact animals; “5 Gy”—isolated X-ray irradiation; “Evo”—drug monotherapy; “Evo 5 Gy”—combined treatment.

As illustrated in Figure 5, the statistical significance profile and cell accumulation patterns are critically dependent on both the animal strain and the specific tissue type. Direct credible synergy (a hyper-additive effect) was observed in the spleen of nude mice. Specifically, isolated treatments (“C” and “Evo”) did not significantly alter the cell percentage (group a), whereas isolated irradiation “5 Gy” and combined exposure “Evo 5 Gy” led to a pronounced increase in *CD45+* cell content (group b). Under combined treatment, an exploratory synergistic shift of 18% was observed (posterior power 69%). In the tumor tissue of C57Bl/6 mice, the point estimate for the interaction was 12%; however, according to the plot (Figure 5), the combined application of “Evo 5 Gy” did not statistically differ from the effects of isolated “5 Gy” irradiation or “Evo” monotherapy (all three experimental groups share the lowercase letter index b). The posterior power for this compartment was determined to be 47%. In the peripheral blood (PBMC) of nude mice, a credible decrease in *CD45+* cells was recorded in the complete absence of synergy. The isolated effects of radiation and the drug allowed these groups to be classified into an intermediate cluster b, whereas the combined “Evo 5 Gy” treatment led to an even more pronounced drop, forming an isolated cluster c. Despite the visual decline, a formal interaction contrast analysis demonstrated that this effect was strictly additive with an estimated transitional power of 45%. The tumor tissue of nude mice proved entirely unreactive to all types of therapeutic intervention; all groups remained at a stably low baseline near zero and were marked by a single index a (Figure 5). The estimated interaction effect was 3% with posterior power 14%, indicating no credible interaction.

These results indicate that the combined effect of evolocumab and radiation is not universal but is tissue- and strain-specific. The most pronounced biological response is localized in the spleen of immunodeficient nude mice (Figure 5). The fact that in wild-type mice (C57Bl/6) the spleen remained completely unresponsive to therapy (all groups belong to cluster a), and in nude mice, it demonstrated a hyperadditive response (transition to cluster b when factors are combined), indicates a deep involvement of innate immunity mechanisms or compensatory proliferation. Since nude mice are depleted of mature T-lymphocytes, the observed synergistic jump in the spleen may be due to the selective accumulation of myeloid-derived cells or NK cells activated by the “Evo 5 Gy” combination in the absence of T-mediated suppression. In contrast, analysis of tumor tissue demonstrates an inverse dependence on the host phenotype. In C57Bl/6 mouse tumors, radiation and the drug significantly reduce or reorganize the cell pool (transition from control a to experimental cluster b in Figure 5), but the combined effect does not provide an additional advantage over monotherapy, demonstrating a plateau in efficacy. In nude mouse tumors, the combined effect is completely suppressed (the effect is “frozen” at zero). This suggests that a preserved and functionally active T-cell adaptive immune system is necessary for even a basic therapeutic response in tumor tissue. We observed similar effects for the Ly6G+ granulocytic cell line, CD11b+ monocytic cell line, and F4/80+ macrophage populations; the results for these cell lines are shown in Supplement Figure S2. For human LUAD tumors in nudes and allograft tumors in C57Bl/6 mice, a significant decrease in neutrophils was observed in tumor tissue in the treatment groups (Figure S2A,B). CD11b+ monocytes were more abundant in the experimental combination treatment group (Figure S2C,D), and the same effect was observed for the tumor macrophage population in C57Bl/6 mice but not in nudes (Figure S2E,F). Nevertheless, the data obtained from these pilot experiments demonstrate the viability of further exploring the combined effects of the “Evo” drug and irradiation across both allograft and xenograft translational models.

## 3. Discussion

The present study represents the first investigation of a combined therapeutic approach integrating PCSK9-it with neutron irradiation. This novel strategy was evaluated in both in vitro and in vivo models, utilizing the human non-small cell lung carcinoma cell line A549 and the murine Lewis lung carcinoma (LLC1) model. These two models are complementary rather than directly equivalent. They differ in tumor origin, host immune status, species background, and tumor biology. We therefore did not make direct biological comparisons between them. Instead, the two-model design was used to test whether the observed effects are reproducible across different immune contexts. The PERMANOVA and Bayesian analyses were specifically chosen to separate the contributions of mouse strain, tissue type, and treatment, and to identify model-specific versus shared responses. Research demonstrates that STK11-deficient LUAD cells are resistant to advanced radiation therapies using particles like protons and neutrons. This serine-threonine kinase is a popular model for targeting therapy in lung cancer, which has been published in a few recent works [20,21]. An important limitation is that matched STK11 WT controls were not included across all key experiments. A549 cells naturally lack functional STK11 and carry additional genetic alterations, whereas LLC1 cells were genetically modified to introduce an STK11 mutation. Therefore, the observed phenotype should be attributed to STK11-deficient LUAD models rather than exclusively to STK11 loss. Future studies with isogenic WT controls are needed to determine which responses are specifically driven by STK11 deficiency. Also, neutron therapy had the greatest effect on reducing tumor cells survival [22].

Particle therapy and combination therapy with evolocumab were shown to reduce the Ki67 index and the expression of pro-tumor markers (FAP α, CD206). Evolocumab suppressed the radiation-induced increased Ki67 index, as well as altered immune cell populations in the tumor microenvironment. The effects of radiation on tumor cell proliferation are ambiguous. On the one hand, radiation-induced DNA damage activates cell cycle checkpoints, leading to cell cycle disruption. On the other hand, radiation can also induce ordinary cancer cells to acquire a cancer stem cell-like phenotype, which not only exhibits high radioresistance but also promotes tumor proliferation [23]. It has long been demonstrated, both in experimental in vivo models and in patients with carcinoma, that Ki-67-positive tumor cells increase during radiation therapy. In discussing these findings, it is crucial to consider that stress-associated tumor responses may involve dynamic shifts in cellular states rather than the modulation of a single proliferative pathway. For example, in mouse xenografts of squamous cell carcinoma, irradiation at a dose of 15 Gy led to a gradual increase in the Ki67 index from 35 ± 5% to 56 ± 5% on the 5th day after irradiation. It was hypothesized that quiescent cells, which exhibit maximum radioresistance in the G0 phase of the cell cycle, might be recruited into the proliferative cycle as a consequence of irradiation [24]. Recent studies have demonstrated that epithelial-state remodeling drives cancer progression under stress-related conditions. Cancer cell populations employ reversible radiation-persistence via phenotypic plasticity, signaling pathway rewiring, epithelial–mesenchymal transition, polyploidization/depolyploidization, and other specific mechanisms to survive radiotherapy and repopulate the tumor [25,26]. For example, ionizing radiation is thought to induce activation of cytoplasmic signaling cascades such as the phosphatidylinositol 3-kinase (PI3K)/protein kinase B (AKT) signaling pathways [27,28,29], RAS/mitogen-activated protein kinase/nuclear factor κB (NF-κB) [30,31], signal transduction pathways and activators of transcription (STAT) and phospholipase C [32]. Another mechanism involves the influence of senescent cells, the appearance of which was induced by radiation therapy: a complex of SASP molecules (cytokines, chemokines, growth factors and proteases) stimulates mitosis, angiogenesis and interstitial recombination in residual tumor tissues after radiation therapy [33]. We demonstrate that evolocumab suppresses the radiation-induced increase in the Ki-67 index in both xenograft and allograft lung cancer models. Similar results have been previously shown only for normal cells under stress conditions, namely, for stimulated MOVAS mouse aortic smooth muscle cells; evolocumab suppressed PDGF-BB-induced MOVAS cell proliferation [34].

Previously, synergy between immunotherapy and radiation has been shown for other types of tumors [35,36,37]. Our initial hypothesis was a potential PCSK9 inhibitor-induced switch of the tumor microenvironment from a “cold” to a “hot” phenotype [38,39], that is, the attraction of immune cells. Unexpectedly, we observed the opposite effect: in the tumor microenvironment, evolocumab led to a decrease in immune cells which may be associated with direct damage to lymphocytes in the tumor microenvironment; impaired chemotaxis due to vascular damage; and the immunosuppressive effect of radiation due to the release of TGF-β and IL-1. These potential explanations should nevertheless be interpreted cautiously, because changes in cell populations and protein-marker levels do not by themselves establish the underlying functional mechanisms. RNA processing and post-transcriptional regulation can also influence cellular responses to stress [40], highlighting the need for further mechanistic studies to clarify the effects observed in our models. The observed decrease in both CD206+ and CD86+ macrophages suggests a general effect on myeloid infiltration rather than a specific M1/M2 polarization shift. Overall, the obtained results confirm that the combination of PCSK9 inhibition and neutron irradiation modulates the tumor immune microenvironment in a strain- and tissue-specific manner. These changes do not represent a straightforward shift toward an immune-active phenotype; functional immune assays are required to determine whether they alter antitumor immunity.

## 4. Materials and Methods

### 4.1. Cell Lines and Obtaining of Mutant Cell Line

A549 cells naturally carry a homozygous loss-of-function mutation in the STK11 gene. The native LLC1 (Lewis Lung Carcinoma) cell line has a wild-type version of the STK11 gene. Cells were kindly provided by Professor Anna Malashicheva from the Institute of Cytology of the Russian Academy of Sciences. Both cell lines were cultured in DMEM/F12 medium containing 10% fetal bovine serum (Himedia, Maharashtra, India) supplemented with 2 mM L-glutamine, 50 U/mL penicillin, and 50 mg/mL streptomycin (PanEco, Moscow, Russia). STK11-Mut was obtained by inoculation using the CrispCas9 system in LLC1 as described previously [15] with sgRNA and FACS.

### 4.2. Hadron Therapy of Cells

Evolocumab (Repatha 140 mg/mL, AMGEN EUROPE, Breda, The Netherlands) was added to experimental wells at concentrations of 0.1 mg/mL, 0.5 mg/mL, and 1.0 mg/mL; control wells were not supplemented with inhibitor. Next day cells were harvested from a 75 cm^2^ mat surface using trypsin/EDTA and counted using a LUNA-II automated cell counter. They were then transferred to cryovials for further transportation and irradiation. LUAD cell lines were irradiated with protons, neutrons, and photons at a dose of 2 Gy using appropriate facilities at the A.F. Tsyb Medical Radiological Research Center in Obninsk, a branch of the National Medical Research Center of Radiology of the Russian Ministry of Health. An NG-14 neutron generator (developed at the N.L. Dukhov All-Russian Research Institute of Astronomy, Moscow, Russia) was used as a neutron source. A Rokus-AM gamma-therapy system (JSC Ravenstvo, Saint Petersburg, Russia) was used for photon therapy, and a Prometheus proton complex (JSC ProTom, Protvino, Moscow Region, Russia) was used for proton therapy.

### 4.3. CCK8 Viability Assay

Cells pre-irradiated with neutrons, protons, and photons were plated at 10^4^ cells per well in 100 µL of growth medium in 96-well plates. Cell viability was measured daily from day 1 through day 5 after irradiation using the Cell Counting Kit-8 (CCK-8) (Servicebio, Wuhan, China) according to the manufacturer’s protocols. Results were detected on a FlexA-200HT plate reader (Allsheng, Zhejiang, China).

### 4.4. Colony Formation

Cells were plated in 100 mm Petri dishes at a seeding concentration of 10,000 cells and grown at 37 °C and 5% CO_2_. After 14 days, colony formation was assessed under a light microscope. After fixation, the colonies were stained with Harris hematoxylin solution for 15 min, washed with tap water, and then dried at room temperature. The resulting colonies were counted, and statistical data were processed.

### 4.5. Real-Time PCR

Total RNA was isolated using MagZol reagent (Magen, Guangzhou, China) according to the manufacturer’s protocol. Reverse transcription was performed with 0.5 µg of total RNA per reaction using the MMLV-RT kit (Eurogen, Moscow, Russia) at 42 °C for 40 min. Real-time PCR mixtures were prepared in triplicate in a 20 µL reaction volume containing 100 ng of cDNA, 0.3 µM oligonucleotide primers, and 4 µL of 5xSybrGreen-mix (Eurogen, Moscow, Russia). Real-time PCR was performed in a DTPrime 5 thermocycler (DNA-Technology LLC, Moscow, Russia) using the following PCR program: an initial step at 95 °C for 5 min, followed by 30 cycles of denaturation at 94 °C for 15 s, annealing at 62 °C for 10 s, and elongation at 72 °C for 20 s. Relative expression levels of the studied genes were calculated as 2^−ΔCt^, where deltaCt was equal to the average Ct of the genes minus the average Ct of the glyceraldehyde-3-phosphate dehydrogenase (GAPDH) gene.

### 4.6. Annexin V/Propidium Iodide Cell Viability Assay

The Annexin V/Propidium Iodide assay was performed using the TransDetect Annexin V-FITC/PI Apoptosis Detection Kit (TransGen, Beijing, China) according to the manufacturer’s protocol. At least 30,000 cells were analyzed in each measurement. The analysis was performed on a LongCyte C3111 analyzer (Challenbio, Beijing, China) with subsequent processing in FlowJo X™ Software (BD Biosciences, Milpitas, CA, USA). 4.14 Data were analyzed using FlowJo X software (BD Biosciences, Milpitas, CA, USA). Data was presented in FlowJo X software to analyze populations of immune cells.

### 4.7. Experimental Animals

The study involved laboratory mice of two strains: immunodeficient Balb/c nude males and inbred C57Bl/6 males. The former were used to create a xenogeneic tumor model, while the latter were used to create a syngeneic tumor model, respectively. The mice averaged 30–33 g in weight and were 3–4 months old. The animals were maintained in conditions optimal for each mouse strain, with constant access to water and food. The animals were housed in plastic cages at a temperature of 22 ± 1 °C under a 12-h light/12-h dark cycle, with lighting on from 6:00 a.m. to 6:00 p.m. They had free access to standard rodent feeder and water. The conditions for keeping laboratory animals complied with the 1985 International Guidelines for Biomedical Research on Animals, the rules for laboratory activities in the Russian Federation (Order of the Ministry of Health of the Russian Federation dated 6 September 2003 No. 267) and the Law on the Protection of Animals from Cruelty, Chapter V, Articles 10, 4679-GD dated 1 December 1999.

### 4.8. In Vivo Tumor Modeling

STK11-deficient A549 and LLC1 cell suspensions in saline (PanEco, Moscow, Russia) were injected subcutaneously into the right and left flank of nude and C57Bl/6 male mice, respectively. The procedure was performed using 1 mL syringes. Each mouse received 100 μL of the cell suspension. The cell concentration was 4 million/mouse for nude and 3.5 million/mouse for C57Bl/6. Two days after injecting the mice with the cell suspensions, they were examined: they were weighed on an analytical balance, and the tumor volume was calculated in mm^3^. For this purpose, the formula was used: Tumor V = a × b × c, where a is the tumor length in mm, b is the tumor width in mm, and c is the tumor height in mm. Weight was measured in grams, accurate to decimal places. Measurements were taken twice a week.

### 4.9. Hadron Therapy of Tumors

After the tumor volume reached 100 mm^3^, the mice were irradiated with fast neurons. Neutron irradiation was performed in Obninsk at the A.F. Tsyb Medical Radiological Research Center, a branch of the National Medical Research Center of Radiology of the Russian Ministry of Health, at a dose of 5 Gy. A Rosatom NG-14 neutron generator was used as the neutron source. The animals were then kept in the animal holding room of the Research Institute of Molecular and Cellular Medicine at RUDN University and were withdrawn from the experiment after a week.

### 4.10. Evolocumab PCSK-9 Inhibition Therapy

Repatha 140 mg/ml (Amgen, Thousand Oaks, CA, USA), containing evolocumab, was used as a PCSK9 (proprotein convertase subtilisin/kexin type 9) inhibitor. The drug is available as a solution for subcutaneous administration. The drug was administered subcutaneously in the back using 1 mL syringes (Figure 1). The needle was inserted parallel to the skin. Each mouse was injected with 100 μL of the drug at a concentration of 10 mg/kg once a week.

### 4.11. Tissue Isolation

Animals were anesthetized with Vesotil 100 (VETSTEM, Moscow, Russia) at a dose of 5 mg/mL. Blood was collected for subsequent mononuclear cell isolation: a chest incision was made, and blood was collected directly from the heart with a pipette and placed in a tube containing 10 U heparin solution (MEZ, Moscow, Russia). In total, 5 mL of 1x erythrocyte lysis buffer (Milteniy Biotech, Rhineland, Germany) was added to 500 μL of collected blood according to the manufacturer’s protocol. The pellet was resuspended in 500 μL of Hank’s solution (PanEco, Moscow, Russia) and supplemented with 500 μL of 4% PFA solution (PanEco, Moscow, Russia). The samples were stored at 4 °C. To carry out spleen removal for subsequent cell suspension preparation and flow cytometry, the spleen was excised using surgical scissors and tweezers and placed in a transport medium—Hank’s solution (PanEco, Moscow, Russia). The spleen was minced mechanically and passed through a 70 μm nylon sieve. Erythrocytes were lysed according to the protocol described above. Tumor tissue was collected and divided into 3 parts: for immunohistochemistry, RNA extraction, and preparation of a cell suspension for flow cytometry. The tumor was excised using surgical scissors and tweezers. The tumor was divided into 4 parts using a scalpel. The first part of the tumor tissue (for subsequent histological and immunohistochemistry assay) was washed in PBS 1x (Servicebio, Wuhan, China), embedded in Tissue-Tek O.C.T. Compound (Sakura Finetek, Tokyo, Japan), and frozen at −80 °C. Samples were also stored at −80 °C. The second portion of tumor tissue (for RNA extraction) was placed in MagZol solution (Magen, Guangzhou, China) and stored for 24 h at −20 °C, then at −40 °C until RNA extraction. The third portion of tumor tissue (for cell suspension preparation and subsequent flow cytometry assay) was placed in Hank’s solution (PanEco, Moscow, Russia). Tumor cell suspensions were prepared using a DSC-400 cell dissociator (RWD, Shenzhen, China) with the High Activity Tumor Tissue Enzymatic Digestion Kit for Mouse (RWD, Shenzhen, China) according to the manufacturer’s protocol. The fourth portion of tumor tissue was frozen in liquid nitrogen for subsequent Western blot analysis.

### 4.12. Histological Sectioning and Staining

Tumor tissue was frozen in Tissue-TEK O.C.T. Compound (Sakura Finetek, Tokyo, Japan). Sections were obtained on a Minux FS800 Auto cryostat (RWD, Shenzhen, China), fixed in ice-cold methanol, and stained with 1% hematoxylin. After rinsing with water, the sections were stained with eosin. Dehydration was performed through several ethanol series of varying concentrations (70%, 80%, 95%, 100%), then cleared with xylene and mounted in CV Mount (Leica Microsystems, Wetzlar, Germany). The prepared sections were analyzed using a Leica DMI8 microscope (Leica Microsystems, Wetzlar, Germany).

### 4.13. Immunohistochemical Analysis

Tumor tissue fragments were transferred to foil molds filled with Tissue-Tek O.C.T. Compound (Sakura Finetek, Tokyo, Japan), frozen at −80 °C until the mounting medium was completely solidified and then stored at −20 °C until use. Cryosections (7 µm thick) were prepared using a Dakewe 6250 cryotome (Dakewe, Shenzhen, China) using Super Frost Gold glass (Leica Microsystems, Wetzlar, Germany). Cryosections were stained with anti-Ki67 antibodies (1:200, ab16667, Abcam, Cambridge, UK) and ABflo ^®^ 555-conjugated antibodies (1:200, AS058, Abclonal, Wuhan, China). Cell nuclei were counterstained with DAPI (Sigma Aldrich, St. Louis, MO, USA). Images were captured using a Leica DM 4000 B upright fluorescence microscope and LAS AF v. 3.1.0 build 8587 software (Leica Microsystems, Wetzlar, Germany). Cryosections were prepared at two levels for each animal, with six randomly selected fields of view being analyzed for each. Two independent researchers used Adobe Photoshop v. 22.0.1 (Adobe, San Jose, CA, USA) to calculate the proportions of Ki67+ and Ki67- cells.

### 4.14. Flow Cytometry

In total, 10^6^ lung stromal cells were incubated in 100 µL of Rinsing Solution (Miltenyi Biotec, Rhineland, Germany) with primary antibodies at 4 °C for 1 h. After incubation, cells were washed in PBS, resuspended in 0.5 mL PBS, and then transferred to tubes for analysis on a MACSQuant 10 flow cytometer (Milteniy Biotech, Rhineland, Germany). Data were analyzed using FlowJo software (BD Biosciences, Milpitas, CA, USA). Ten thousand cells were analyzed in each measurement. The population of interest was determined using a front scatter (FSC) and side scatter (SSC) plot, excluding debris. Next, the CD45+ cell population, common to all leukocytes and macrophages, was identified using the SSC-CD45 dot plot. To analyze the number of cell populations positive for a marker of interest, we identified the positive population in the SSC and corresponding marker dot plots. The gate for positive cells was set using an unstained sample. Data was presented in Flow Jo X software to analyze populations of immune cells.

### 4.15. Western Blotting

Protein lysates were analyzed by Western blotting, using electrophoresis in 12% polyacrylamide gel under denaturing conditions. The proteins were then transferred to a PVDF membrane for 1.5 h at a constant current of 250 mA in an ethanol-containing buffer. To prevent nonspecific protein binding, the membrane was incubated in TBST buffer (50 mM Tris-HCl, pH 8.0, 150 mM NaCl, 0.05% Tween 20) containing 2% bovine serum albumin for 1 h at room temperature. Then the membrane was incubated overnight with primary antibodies Anti-Fibroblast activation protein (Abcam, ab28244, 1/700), anti-GAPDH (HyTest, Cat. No. 5G4, clone 4G5, 1/5000), Anti-CD86 (Santa Cruz, sc-28347, 1/700), Anti-Mannose Receptor (CD206) (Abcam, ab64693, 1/1000), goat anti-rabbit IgG (Abcam, ab6721, 1/1000), goat anti-mouse IgG (Abcam, ab6789, 1/2000), for which the concentration of each antibody was selected. The membrane was then washed with TBST buffer to remove primary antibodies, and secondary goat anti-rabbit or anti-mouse IgG antibodies conjugated with horseradish peroxidase (Jackson ImmunoResearch) were added. The membranes were then treated with SuperSignal West Pico chemiluminescence detection solution, and the signals were detected on a ChemiDoc instrument (Bio-Rad, Hercules, CA, USA). The signal was detected on a Fusion FX system (Vilber Lourmat, Collégien, France) using Fusion Software v18.02 (Vilber Lourmat, Collégien, France).

### 4.16. Statistical Analysis

Statistical data processing was performed using the GraphPad Prism 8 program (GraphPad Software, San Diego, CA, USA). The Shapiro–Wilk test was used to test the nature of the sample distribution. One-way analysis of variance (ANOVA) followed by Tukey’s HSD test was used to identify differences between multiple normally distributed groups. The nonparametric Kruskal–Wallis analysis of variance followed by Dunn’s test was used to calculate statistical differences for non-normal distributions. Differences were considered statistically significant when *p* < 0.05 for all tests. Statistical analysis and data visualization for FC were performed using the R environment (v. 4.5.2) [41] with the core integration of ggplot2 (v. 4.0.3) [42], multcomp (v. 1.4-30) [43], and the tidyverse framework (v. 2.0.0) [44].

#### 4.16.1. Three-Factor Tissue-Specific Profile Analysis and PCoA Ordination

The effects of mouse genetic strain (Mice), therapeutic intervention (Sample), and their potential interference patterns were evaluated via marginal permutational multivariate analysis of variance (PERMANOVA) using the adonis2 function from the vegan package (v. 2.7-5) [45] with 999 free permutations. Multivariate homogeneity of group dispersions was verified using the betadisper test. For a comprehensive, systems-level assessment of cellular population shifts across the three investigated compartments (peripheral blood PBMC, Spleen, and Tumor), individual animal profiles were projected into a multivariate feature space characterized by a 2 × 3 × 4 factorial design (Mice × Tissue × Sample). To visualize multivariate CD45+ cell-count interactions under varying experimental factors, Principal Coordinate Analysis metric multidimensional scaling (PCoA, cmdscale function) was applied. The pairwise biological distance matrix between individual samples was computed based on Euclidean distances.

#### 4.16.2. Three-Factor Robust Bayesian Analysis

To assess the individual and combined effects of the drug (“Evo”) and X-ray irradiation (“5 Gy”) on the percentage of CD45+ cells, a three-factor robust Bayesian linear model was constructed using Markov Chain Monte Carlo (MCMC) methods. Fixed effects included mouse strain mice (C57Bl/6, Nude), type of tissue (PBMC, Spleen, Tumor), and the treatment regimen sample (Control “C”, “5 Gy”, “Evo”, “Evo 5 Gy”), alongside all possible interaction terms. Given the heteroscedastic nature of the data and the potential presence of isolated outliers, a Student’s $t$-distribution (family = student()) was selected as the likelihood function. Parameter estimation was performed within the Stan computational environment via the cmdstanr interface (v. 0.8.0) [46] within the brms package (v. 2.23.0) [47]. For each parameter, 4 independent MCMC chains were run for 4000 iterations each, with the first 2000 iterations discarded as warmup. The robustness of the fit was verified using the shape parameter while sensitivity to outliers was evaluated via leave-one-out cross-validation (LOO-CV, loo_compare function). Therapeutic synergy was evaluated using interaction contrasts derived from posterior group means implemented via emmeans (v. 2.0.3) [48] and dplyr (v. 1.2.1) [49]. The synergy interaction effect was defined as follows: Synergy = (Evo 5 Gy − 5 Gy) − (Evo-C). An effect was deemed statistically credible if its 95% highest posterior density (95% HPD) interval excluded zero (0). Additionally, a posterior power simulation analysis (1000 replicates) was conducted to evaluate the reliability of detecting synergistic shifts given the current sample sizes.

## 5. Conclusions

As a result of the study, models of STK11-mutant adenocarcinoma of the lung were developed both in vitro and in vivo to evaluate the combination therapy PCSK9 inhibitor—evolocumab and neutron therapy. The study revealed suppression of radio-induced proliferation under the influence of evolocumab, as well as changes in immune-cell populations in the tumor microenvironment. These are associations and do not prove functional regulation; functional immune assays are required to determine whether these changes alter anti-tumor immunity. Thus, the main characteristics of the effects of hadron therapy and its impact on the immune response in the presence of PCSK9-it has been established. However, further research is needed to fully understand the mechanisms underlying the effectiveness of this approach and to optimize its use in clinical settings. Additionally, larger-scale studies are required to confirm the translational potential of this strategy in human patients. Despite these challenges, the results of this investigation provide a strong foundation for future research in this area. The development of novel cancer treatments is a critical need in oncology, and the combination of advanced radiation therapies with targeted therapies offers a promising avenue for addressing this need.

## Data Availability

The original contributions presented in this study are included in the article and/or Supplementary Material. Further inquiries can be directed to the corresponding author.

## Supplementary Materials

The following supporting information can be downloaded at https://www.mdpi.com/article/10.3390/ijms27219778/s1.

## Abbreviations

The following abbreviations are used in this manuscript:

PCSK9	Proprotein convertase subtilisin/kexin type 9
LLC1	Lewis lung carcinoma 1
STK11/LKB1	Serine/threonine kinase 11, Liver kinase B1
LUAD	Lung adenocarcinoma
PCSK9-it	PCSK9 inhibition therapy
