# Supplementary material for: Combined Effect of Hadron Therapy and PCSK9 Inhibition Therapy on the Immune Response in STK11-Deficient Lung Adenocarcinoma
Source: Int J Mol Sci. 2026 Oct 30;27(21):9778. doi: 10.3390/ijms27219778 (PMC13763336; doi:10.3390/ijms27219778)
Supplement: Supplementary file 1 [file ijms-27-09778-s001.zip › Supplementary Table S1.pdf]

Supplement Table S1. Flow cytometry antibodies, all anti-mouse.

**Antibody**	**Manufacturer**	**Cat. №**	**Lot**	**Clone**
CD45 PerCP	Scientific Research Antibody	DL21684F	ADL2022102715	
CD11b Violet 450	Elabscince	E-AB-F1081Q	AF20017	M1/70
CD45 APC	Miltenyi Biotec	130-110-798	5221200255	REA737
Ly-6G PE	Miltenyi Biotec	130-102-392	5180920062	
F4/80 PE	Miltenyi Biotec	130-116-499	5210609428	REA126
